# *Cryptosporidium parvum*, *Cryptosporidium ryanae*, and *Cryptosporidium bovis* in samples from calves in Austria

**DOI:** 10.1007/s00436-020-06928-5

**Published:** 2020-10-15

**Authors:** Katharina Lichtmannsperger, Josef Harl, Katharina Freudenthaler, Barbara Hinney, Thomas Wittek, Anja Joachim

**Affiliations:** 1 University Clinic for Ruminants, Department for Farm Animals and Veterinary Public Health, University of Veterinary Medicine Vienna, Wien, Austria; 2 Institute of Pathology, Department of Pathobiology, University of Veterinary Medicine Vienna, Wien, Austria; 3 Institute of Parasitology, Department of Pathobiology, University of Veterinary Medicine Vienna, Wien, Austria

**Keywords:** Protozoal infection, *gp60*, *18S*, Fecal consistency

## Abstract

Fecal samples of 177 calves of up to 180 days of age with diarrhea from 70 farms in Austria were examined to obtain information on the occurrence of *Cryptosporidium* species. Initially, all samples were examined by phase-contrast microscopy. *Cryptosporidium*-positive samples (55.4%; *n* = 98) were screened by *gp60* PCR, resulting in 68.4% (*n* = 67) *C. parvum*–positive samples. The remaining 31 *gp60*-PCR-negative and the phase-contrast microscopy negative samples (*n* = 79) were screened by PCR targeting a 700 bp fragment of the *18S* rRNA gene. Sequencing of the PCR products revealed the presence of *C. parvum* (*n* = 69), *C. ryanae* (*n* = 11), and *C. bovis* (*n* = 7). The latter two species have never been described in Austria. *C. parvum–*positive samples were genotyped at the *gp60* gene locus, featuring four subtypes (IIaA15G2R1, IIaA21G2R1, IIaA19G2R1, IIaA14G1R1). The most frequently detected subtype IIaA15G2R1 (*n* = 52) was present in calves from 30 different farms. IIaA14G1R1 (*n* = 5) occurred on a single farm, subtype IIaA21G2R1 (*n* = 4) on two farms, and subtype IIaA19G2R1 (n = 4) on three farms. The results confirm the widespread occurrence of zoonotic *C. parvum* in diarrheic calves.

## Introduction

To date, 38 *Cryptosporidium* species are known, of which four—*C. parvum*, *C. bovis*, *C. ryanae*, and *C. andersoni*—can be found in cattle. *Cryptosporidium parvum* and *C. bovis* are responsible for over 90% of bovine infections (Feng et al. 2018; Widmer et al. 2020). *Cryptosporidium parvum* is associated with diarrhea in neonatal calves and intra-herd prevalence extends up to 100% (Avendaño et al. 2018; Holzhausen et al. 2019; Thompson et al. 2017). *Cryptosporidium bovis* and *C. ryanae* are primarily found in the feces of post-weaned calves and *C. andersoni* in the abomasum of adult cattle (Ryan et al. 2014). Nevertheless, *C. ryanae* and *C. bovis* were isolated from pre-weaned diarrheic and healthy calves in certain areas of Sweden, China, and Sudan (Silverlås et al. 2010; Taha et al. 2017; Wang et al. 2011). Human cryptosporidiosis is primarily caused by *C. parvum* and *C. hominis*, and diarrheic and healthy calves are considered major reservoirs for human infections (Razakandrainibe et al. 2018; Ryan et al. 2014). *Cryptosporidium* oocysts are microscopically indistinguishable due to their similar size and shape. Therefore, molecular methods are indispensable for species differentiation (Ryan et al. 2014). Previous investigations focusing on the typing of *C. parvum* by sequencing a section of the *60-kD glycoprotein* (*gp60*) gene in calves primarily report the occurrence of the subtype families IIa and IId with some geographical differences (Feng et al. 2018; Ryan et al. 2014). Subtype family IIa dominates in industrialized nations such as Italy (Díaz et al. 2018), the USA (Xiao et al. 2007), New Zealand (Abeywardena et al. 2012), and Austria (Lichtmannsperger et al. 2019). Subtype family IId was commonly reported from less industrialized countries (Ryan et al. 2014) such as Sudan (Taha et al. 2017), Malaysia (Muhid et al. 2011), Egypt (Amer et al. 2013), and China (Wang et al. 2011). Subtype IIaA15G2R1 has been described as the predominant subtype in symptomatic and asymptomatic calves worldwide (Feng et al. 2018; Holzhausen et al. 2019).

Differentiation of *Cryptosporidium* on species and subtype level is apparently lacking in Austria. The aim of this study was to determine the occurrence of different *Cryptosporidium* species and genotypes in calves with diarrhea less than 180 days of age. It was hypothesized that besides *C. parvum*, other *Cryptosporidia* species occur in feces of diarrheic calves in Austria.

## Material and methods

### Sample collection and microscopic examination

Farmers and veterinarians from all over Austria were contacted and asked to participate in the study. In total, 177 calves with diarrhea originating from 70 farms were included. The fecal samples used in this investigation were collected during the study on the occurrence of *C. parvum* and *Giardia intestinalis* in diarrheic calves in Austria (Lichtmannsperger et al. 2019). Samples were collected per rectum during a farm visit. All calves younger than 180 days of age with diarrhea (soft, liquid, or watery feces) were included and sampled once by the first author or the local veterinarians. The samples were transferred to the Institute of Parasitology at the University of Veterinary Medicine Vienna for immediate diagnostics. All samples were screened for *Cryptosporidium* spp. by phase-contrast microscopy (PCM) as described previously (Lichtmannsperger et al. 2019). In brief, sample purification was performed using the sodium-acetate-acetic formalin (SAF) method, and the pellet was resuspended in phosphate-buffered saline. The suspension was filled into the chamber of a disposable hemocytometer, and oocysts were counted using PCM with 200-fold magnification. The number of oocysts was given in oocysts per gram feces (*opg*).

### DNA extraction and PCR for genotyping

DNA was extracted from all diarrheic fecal as described previously (Lichtmannsperger et al. 2019).

For the detection of all *Cryptosporidium* spp. except *C. parvum*, a nested PCR protocol was implemented to amplify a 700 bp fragment of the nuclear *18S* rRNA gene (*18S*). For genotype analysis of *C. parvum*, a 450 bp section of the *gp60* gene was amplified. The *18S* PCR was performed on all PCM-negative and *gp60*-negative samples. The primers were designed based on complete or almost complete *18S* sequences of various apicomplexan parasites mined from NCBI GenBank (https://www.ncbi.nlm.nih.gov/genbank/). The quality of the primers was tested using AmplifX v.2.0.7. (Nicolas Julien; https://inp.univ-amu.fr/en/amplifx-manage-test-and-design-your-primers-for-pcr) and Primer-BLAST implemented in NCBI GenBank. The primers (Table 1) are specific to the genus *Cryptosporidium* and do not amplify the *18S* of other apicomplexan parasites. The reaction volume (25 μl) contained 1 μl of genomic DNA template, 14.375 μl nuclease free water, 5.0 μl of 5X Green GoTaq® Reaction Buffer (Promega, USA), 2.0 μl of 25 mM MgCl_2_, 0.5 μl of 10 mM dNTP mix, 0.125 μl GoTaq G2® Polymerase (5 U/μl, Promega), and 1.0 μl each of 10 mM oligonucleotide primers (Table 1). For the second PCR round, 0.5 μl template from the previous PCR was used. The cycling protocol for both reactions included an initial cycle of 94 °C for 2 min, followed by 20 (nest 1)/35 (nest 2) cycles of 94 °C for 30 s, 56 °C for 60 s, 72 °C for 60 s, and a final extension of 72 °C for 5 min.

**Table 1:** Primers utilized in nested PCR reactions amplifying sections of the *18S* of *Cryptosporidium* spp. and the *gp60* of *Cryptosporidium parvum* from fecal samples. The *18S* primers and the protocol were designed for this investigation; the implemented protocol for the detection of *gp60* was described previously by Peng et al. (2001)

	Primer	Primer sequence (5′-3′)	Amplicon size (bp)	Annealing (°C)
*18S*	Crypto18S_F1	for: ACATATCATTCAAGTTTCTGACCTATC	766	56
	Crypto18S_R1	rev: TCTCATAAGGTGCTGAAGGAGT		
	Crypto18S_F2	for: CAGCTTTAGACGGTAGGGTATTGG	740	56
	Crypto18S_R2	rev: TAAGGTGCTGAAGGAGTAAGGAAC		
*gp60*	AL3531	for: ATAGTCTCCGCTGTATTC	850	56
	AL3534	rev: GCAGAGGAACCAGCATC		
	AL3532	for: TCCGCTGTATTCTCAGCC	450	60
	AL3533	rev: GAGATATATCTTGGTGCG		

The *gp60* PCRs were carried out on all samples positive by phase-contrast microscopy as described previously (Lichtmannsperger et al.2019). In brief, 1 μl of genomic DNA was used in a 25 μl reaction volume with 13.675 μl of nuclease free water, 5.0 μl of 5X Green GoTaq® Reaction Buffer (Promega), 0.2 μl of 25 mM dNTPs, 3.0 μl of 25 mM MgCl_2_, 0.125 μl of GoTaq® G2 DNA Polymerase (5 U/μl), and 1 μl each of 20 pmol oligonucleotide primers (Table 1). For the second PCR round, 0.5 μl template from the previous PCR was used. The cycling protocol for both reactions included one cycle of 94 °C for 2 min, followed by 30 cycles of 95 °C for 50 s, 56 °C (nest1)/60 °C (nest 2) for 50 s, 65 °C for 60 s, and a final extension of 65 °C for 5 min.

PCR products were subjected to electrophoresis on 2.0% agarose gels and visualized with ultraviolet light (LumiBIS 1.4, DNR Bio-Imaging Systems Ltd., Israel).

### Sequencing of PCR products

Purification and sequencing in both directions was done at LGC Genomics GmbH (Berlin, Germany). The raw forward and reverse sequences (and electropherograms) were carefully checked and aligned with Bioedit v.7.0.8.0 (Hall 1999). Sequences were subjected to BLAST (https://blast.ncbi.nlm.nih.gov/Blast) searches at NCBI GenBank to identify the respective *gp60* and *18S* variants. All sequences were deposited in NCBI GenBank under the accession numbers (*18S*: MT611069–MT611099; *gp60*: MT637080- MT637083).

### Statistical analysis

The data were organized using IBM® SPSS® Statistics Version 24 (IBM, New York, USA). Normal distribution was calculated using the Kolmogorov-Smirnov test. A chi^2^ test was implemented for the comparison of categorical variables (fecal consistency). The mean *C. parvum*, *C. ryanae*, and *C. bovis* shedding was only calculated with samples confirmed by *gp60* or *18S* PCR. The age differences in calves shedding *C. parvum*, *C. ryanae*, and *C. bovis* were analyzed by using a one-way ANOVA and the post hoc Bonferroni correction for multiple testing. Differences were considered statistically significant if *p* ≤ 0.05.

## Results and discussion

One to 10 animals were sampled per farm (median = 2; mean = 2.5). The fecal consistency of the diarrheic calves appeared soft (*n* = 72), liquid (*n* = 82), or watery (*n* = 23). The age ranged from 1 to 164 days (median = 12; mean = 27). The average oocyst shedding (*n* = 98) was 1 × 10^5^ *opg* (range = 3.0 × 10^3^–3.0 × 10^7^; median = 1.0 × 10^6^; SD = 3.0 × 10^6^) (Lichtmannsperger et al. 2019).

**Fig. 1:**
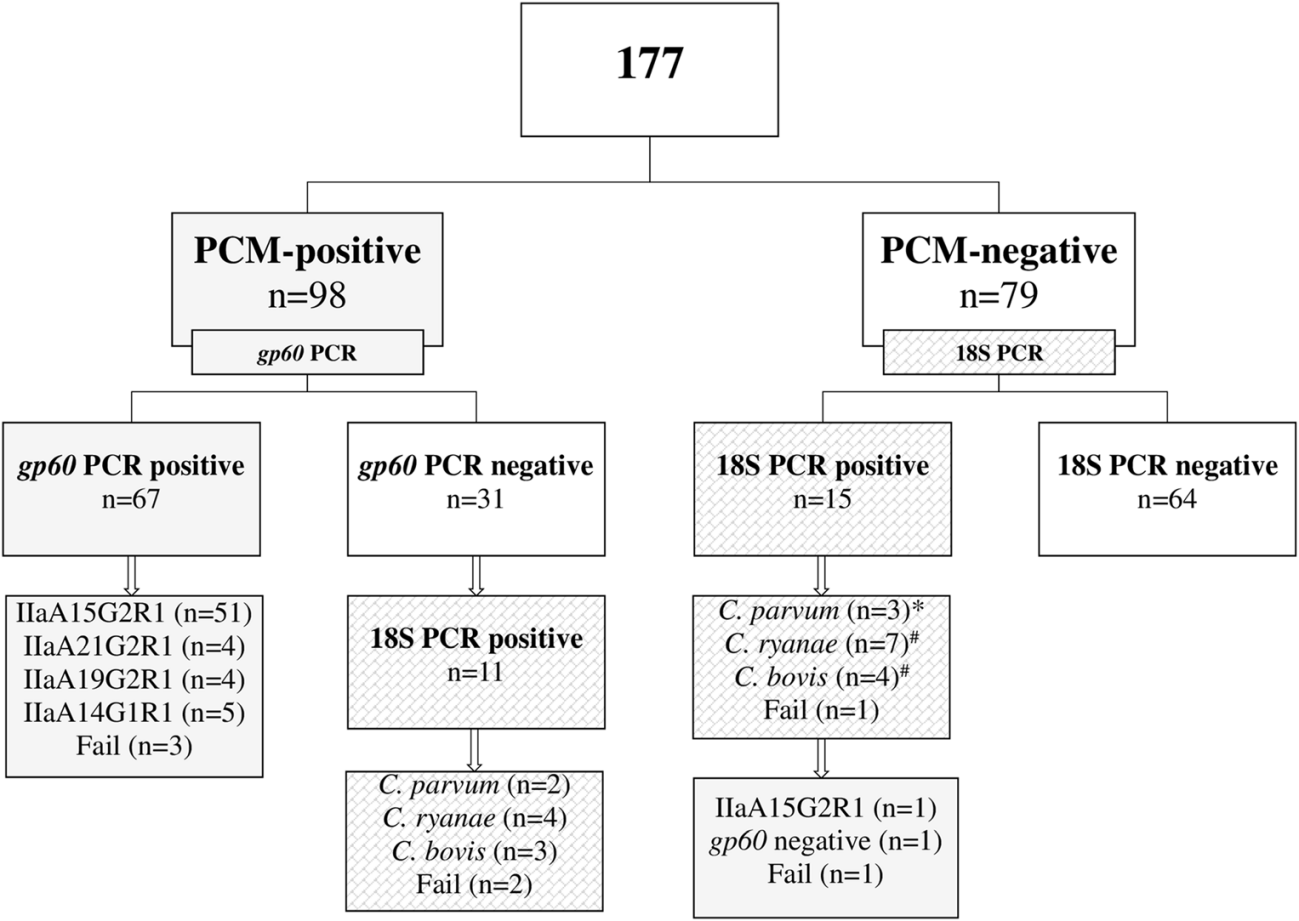
A total of 177 fecal samples from diarrheic calves were screened by phase-contrast microscopy (PCM). Samples were further analyzed by the *C. parvum*–specific 60 kD-glycoprotein PCR (*gp60*) and the *Cryptosporidium* species–specific *18S* PCR. For the determination of *C. parvum* subtypes, the *gp60* locus was used (Fail = sequencing unsuccessful). *Phase-contrast microscopy negative and *18S* positive *C. parvum* samples were further characterized using fragments of the *gp60* gene. ^#^Double infection with *C. bovis* and *C. ryanae* in one sample

Previously, the widespread occurrence of *Cryptosporidium* spp. in diarrheic calves from Austria was described, but without differentiation at the species and subtype levels (Lichtmannsperger et al. 2019). All PCM-positive samples (*n* = 98) were screened using the *gp60* PCR, of which 68.4% (67/98) yielded positive results. The remaining *gp60*-negative samples (*n* = 31) were screened for *Cryptosporidium* spp. using the *18S* PCR assay (see Fig. 1 for details). All *gp60*-positive samples were further sequenced to determine *C. parvum* subtypes. Four subtypes (IIaA15G2R1, IIaA14G1R1, IIaA21G2R1, IIaA19G2R1) were detected. The most frequently detected subtype was IIaA15G2R1 (*n* = 52) which was found on 30 farms. IIaA14G1R1 (*n* = 5) occurred on a single farm, subtype IIaA21G2R1 (*n* = 4) on two farms, and subtype IIaA19G2R1 (*n* = 4) on three farms. Subtype IIaA15G2R1 is the predominant subtype in symptomatic and asymptomatic calves worldwide, which was in accordance with our findings (Feng et al. 2018). A high subtype diversity but endemicity of a single subtype within herds or regions has previously been found in areas where animal movement is limited (Brook et al. 2009; Silverlås et al. 2010). Due to the implemented study design (sample size calculation, randomization), information concerning on-farm prevalence of subtypes is limited. Authors from Sweden reported similar observations of the on-farm-specific occurrence of *C. parvum* subtypes and assume that this was due to the dominating closed herd management systems (Silverlås et al. 2010). Closed herd management systems are common due to the small structured agriculture in Austria, which might be the reason for the similar results.

For the detection of further *C. parvum* species, the *18S* PCR assay was performed on all PCM-negative and all *gp60*-negative samples (Fig. 1). In total, *C. parvum* (*n* = 5), *C. ryanae* (*n* = 11), and *C. bovis* (*n* = 7) were detected. One sample contained both *C. ryanae* and *C. bovis*. The presence of both species in this sample was evident by double peaks in the electropherograms. Since the *18S* section analyzed was of same length in *C. ryanae* and *C. bovis*, the distinction of the two haplotypes was straightforward. Sequence analysis failed in four samples, which could be due to the presence of multiple *Cryptosporidium* strains.

The age of calves positive for *C. parvum* (*n* = 72) ranged from 3 to 127 days (mean = 14.5; media*n* = 10.0), 9 to 126 (mea*n* = 43.5; media*n* = 35.0) for *C. ryanae* (*n* = 10), and 11 to 119 (mean = 64.6; median = 60.0) for *C. bovis* (n = 7). The age of the calves shedding *C. parvum* was significantly lower than from calves shedding *C. bovis* or *C. ryanae* (*p* = 0.00; *p* = 0.022). Between *C. bovis* and *C. ryanae* shedding calves, the age difference was not statistically significant (*p* = 0.866). The average number of *opg* shed by diarrheic calves was 1.7 × 10^6^ (range = 5.0 × 10^3^ to 2.6 × 10^7^; median = 4.4 × 10^5^) for *C. parvum* (*n* = 69), 1.1 × 10^4^ (range: 2.5 × 10^3^ to 2.3 × 10^4^; median = 10^4^) for *C. ryanae* (n = 4), and 1.3 × 10^4^ (range = 2.5 × 10^3^ to 2.8 × 10^4^; median = 1.0 × 10^4^) for *C. bovis* (n = 3). Oocysts of *C. bovis* and *C. ryanae* were shed in lower numbers in comparison to *C. parvum.* However, the number of excreting animals was too low for statistical comparison.

Some authors report a higher prevalence of *Cryptosporidium* spp. when using light microscopy, which is in accordance with our findings (Taha et al. 2017). A potential explanation might be that some of the microscopy-positive samples were wrongly classified due to the morphologically similar appearance of other particles such as yeast spores (Taha et al. 2017).

Genetic characterization of diarrheic fecal samples using the *18S* PCR assay showed the occurrence of *C. ryanae* and *C. bovis* in young diarrheic calves. The youngest animals were 9 and 11 days old, respectively. The results are in accordance with other studies, where *C. bovis* was found in calves from 5 days of age and *C. ryanae* from the second week of live (Åberg et al. 2019; Wang et al. 2011). Considering *Cryptosporidium* PCR results (*gp60* or *18S; n* = 93), the occurrence of *C. parvum* (77.4%), *C. ryanae* (11.8%), and *C. bovis* (7.5%) is comparable to an investigation from the Sudan on young (< 6 months) diarrheic calves, which featured *C. parvum* (73.5%), *C. ryanae* (13.2%), and *C. bovis* (1.8%) (Taha et al. 2017). Additionally, *C. andersoni* was detected, which was not the case in the present study. Since only *gp60*-negative samples were screened with the *18S* PCR assay, the occurrence of *C. ryanae* and *C. bovis* was likely underestimated in the present sample.

*C. parvum* occurred significantly more often (*p* = 0.007) in animals with liquid or watery diarrhea (*n* = 105) versus animals showing softened feces (*n* = 72). *Cryptosporidium ryanae* and *C. bovis* exclusively occurred in animals with soft (*n* = 8) respectively liquid (*n* = 9) fecal consistency; none of the animals showed watery diarrhea. *Cryptosporidium bovis* was discussed as potentially pathogenic in a study in Sweden, where *C. bovis* was found in diarrheic calves as the only pathogen (Silverlås et al. 2013). Another investigation found no association between the presence of diarrhea and *C. bovis* or *C. ryanae* shedding (Åberg et al. 2019).

The results show the common occurrence of the zoonotic species *C. parvum* and the host-specific *C. ryanae* and *C. bovis* in diarrheic calves in Austria. *C. parvum*–infected calves are shedding high numbers of oocysts which leads to severe environmental contamination and further transmission. Young calves suffering from liquid or watery diarrhea must be considered *C. parvum* shedders and therefore have the potential to cause human infection. Due to the implemented methodology, the simultaneous occurrence of *Cryptosporidium* species cannot be excluded. The number of *C. bovis* and *C. ryanae* positive samples was probably underestimated in the examined samples. Molecular methods such as genotype-specific or multiplex PCR procedures should shed more light on the occurrence of coinfections with different *Cryptosporidium* species or genotypes.
